# Predicting the occurrence of mild cognitive impairment in Parkinson’s disease using structural MRI data

**DOI:** 10.3389/fnins.2024.1375395

**Published:** 2024-04-18

**Authors:** Iman Beheshti, Ji Hyun Ko

**Affiliations:** ^1^Department of Human Anatomy and Cell Science, Rady Faculty of Health Sciences, University of Manitoba, Winnipeg, MB, Canada; ^2^PrairieNeuro Research Centre, Kleysen Institute for Advanced Medicine, Health Science Centre, Winnipeg, MB, Canada; ^3^Graduate Program in Biomedical Engineering, Price Faculty of Engineering, University of Manitoba, Winnipeg, MB, Canada

**Keywords:** prognosis, mild cognitive impairment, voxel-based morphometry, Parkinson’s disease, support vector machine, machine learning

## Abstract

**Introduction:**

Mild cognitive impairment (MCI) is a common symptom observed in individuals with Parkinson’s disease (PD) and a main risk factor for progressing to dementia. Our objective was to identify early anatomical brain changes that precede the transition from healthy cognition to MCI in PD.

**Methods:**

Structural T1-weighted magnetic resonance imaging data of PD patients with healthy cognition at baseline were downloaded from the Parkinson’s Progression Markers Initiative database. Patients were divided into two groups based on the annual cognitive assessments over a 5-year time span: (i) PD patients with unstable healthy cognition who developed MCI over a 5-year follow-up (PD-UHC, *n* = 52), and (ii) PD patients who maintained stable healthy cognitive function over the same period (PD-SHC, *n* = 52). These 52 PD-SHC were selected among 192 PD-SHC patients using propensity score matching method to have similar demographic and clinical characteristics with PD-UHC at baseline. Seventy-five percent of these were used to train a support vector machine (SVM) algorithm to distinguish between the PD-UHC and PD-SHC groups, and tested on the remaining 25% of individuals. Shapley Additive Explanations (SHAP) feature analysis was utilized to identify the most informative brain regions in SVM classifier.

**Results:**

The average accuracy of classifying PD-UHC vs. PD-SHC was 80.76%, with 82.05% sensitivity and 79.48% specificity using 10-fold cross-validation. The performance was similar in the hold-out test sets with all accuracy, sensitivity, and specificity at 76.92%. SHAP analysis showed that the most influential brain regions in the prediction model were located in the frontal, occipital, and cerebellar regions as well as midbrain.

**Discussion:**

Our machine learning-based analysis yielded promising results in identifying PD individuals who are at risk of cognitive decline from the earliest disease stage and revealed the brain regions which may be linked to the prospective cognitive decline in PD before clinical symptoms emerge.

## 1 Introduction

Parkinson’s disease (PD), affecting 2%–3% of the population aged 65 and older, is the second-most common neurodegenerative disorder (Poewe et al., 2017). PD is identified by a variety of motor difficulties including stiffness, shaking, and slowness (Sveinbjornsdottir, 2016). PD patients are also at risk of developing non-motor symptoms, such as cognitive impairment, which can have a major effect on healthcare system, the quality of life of the patient and their family (Chaudhuri et al., 2006; Svenningsson et al., 2012). Cross-sectional studies have documented that about 30% of PD patients are associated with dementia, and 20%–25% of them have mild cognitive impairment (MCI) at the time of diagnosis (Aarsland et al., 2005). Longitudinal studies have reported that, on average, 50% of PD patients are at risk of developing dementia within a decade and this likelihood increases with age (Williams-Gray et al., 2013; Aarsland et al., 2017). In PD, cognitive decline is usually indicated by challenges in executive function (such as organizing, planning, and prioritizing tasks), a slower rate of cognitive processing, attention deficits, impairment of compromised visuospatial abilities and working memory (Chaudhuri et al., 2006; Svenningsson et al., 2012). Notably, PD patients with MCI (PD-MCI) are particularly prone to developing dementia (Aarsland and Kurz, 2010; Pedersen et al., 2013; Aarsland et al., 2021). In spite of its widespread occurrence, substantial cognitive problems in the early stages of PD are often not recognized in clinical settings due to the complex nature of cognitive impairment in PD, which affects multiple aspects of cognition (Wyman-Chick et al., 2017). It is thus essential to recognize the basis of cognitive decline in PD and its association with brain structure and function in order to devise effective interventions for individuals with PD.

Neuroimaging techniques are capable of identifying the pathological changes associated with neurodegenerative diseases, including PD (Risacher and Saykin, 2013; Politis, 2014). Magnetic resonance imaging (MRI) is one of the most widely used neuroimaging technique that can provide insights into the structural changes occurring in the brain. Several studies have used MRI to investigate the alterations in brain volume, cortical thickness, and white matter integrity that can be linked to cognitive decline in PD (Beyer et al., 2007; Song et al., 2011; Mak et al., 2014; Gao et al., 2017; Devignes et al., 2021; Li et al., 2022; Zhu et al., 2022). For example, PD-MCI patients have shown significant atrophy in the frontotemporal cortices, thalamus, nucleus accumbens, as well as caudate nucleus compared to the PD patients with healthy cognition (PD-HC) (Zhou et al., 2020). Machine learning technologies have been utilized to develop algorithms that classify PD-MCI vs. PD-HC based on structural T1-weighted (T1w) MRI images, and identified the right anterior entorhinal cortex (BA 34) (Cho, 2019) and right caudate nucleus (Shibata et al., 2022) as most contributive regions for this classification. There have been only a limited number of studies that have investigated the use of machine-learning methods to predict cognitive outcomes in PD before the onset of clinical symptoms, and these studies have primarily relied on clinical data for their analysis (Smith et al., 2021; Harvey et al., 2022).

It is yet unknown if the brain structural changes precede symptomatic cognitive decline. If it does, it will provide us an opportunity to develop a prognostic biomarker, which may be utilized in identifying susceptible individuals for preventive interventions, which include both pharmacological and non-pharmacological approaches targeting modifiable risk factors (Guo et al., 2019). Early identification of individuals who will later develop severer symptoms can significantly reduce societal burdens related healthcare (Perron et al., 2023). Therefore, it is necessary to examine the anatomical distinctions between PD patients with stable healthy cognition and those who initially have stable healthy cognition but later develop MCI, regardless of their clinical differences. This investigation could potentially enhance our understanding of structural brain changes in PD caused by MCI at very early stages.

This research aims to develop an imaging-based biomarker that differentiate PD-HC patients who later developed MCI within 5 years (unstable PD-HC; PD-UHC) from PD-HC who maintained healthy cognitive function from baseline to 5 years (stable PD-HC; PD-SHC). Support vector machine (SVM) classifier was trained using baseline structural T1w MRI, and the Shapley Additive Explanations (SHAP) feature analysis was performed to identify the relevant brain regions for the proposed classifier.

## 2 Materials and methods

### 2.1 Dataset and sample selection

Data used in this study was obtained from the Parkinson’s Progression Markers Initiative (PPMI) as of September 2022. Ethical standards committees had granted approval to all PPMI sites, and all participants had provided written consent to participate. Additional information about the PPMI protocol approvals, registrations, and patient consents can be found on: https://www.ppmi-info.org/.

We acquired a dataset of 373 PD patients, which included their baseline T1w MRI scans, age, sex, educational background, age of symptom onset, and disease duration (time from initial diagnosis to MRI scan), as well as motor and non-motor clinical measurements.

Parkinson’s Progression Markers Initiative T1w MRI scans have been obtained with standardized acquisition parameters on MRI scanners (e.g., Siemens, Philips, and GE) from different sites and 3D volumetric sequences (e.g., IR-FSPGR and MP-RAGE) in the sagittal plane with high resolution, followed by a slice thickness of 1.2 mm or less. More details on the MRI scan acquisition protocol in the PPMI dataset can be found at: https://www.ppmi-info.org/study-design/research-documents-and-sops.

Motor assessment included the Unified Parkinson’s Disease Rating Scale Part III (UPDRS-III total), UPDRS-III (total tremor), and UPDRS-III (total rigidity) (Goetz et al., 2008). Non-motor symptom scores included Montreal Cognitive Assessment (MoCA), Letter Number Sequencing, Epworth Sleepiness Scale (ESS), and Benton Judgment of Line Orientation Score (BJLO) (Schapira et al., 2017). Mood symptoms included the Geriatric Depression Scale (GDS) and the State-Trait Anxiety Inventory (STAI) (Schapira et al., 2017; Bloem et al., 2021). The cognitive status of PD patients was ascertained by evaluating variables labeled “cogstate” and “MCI test score,” as recorded in the PPMI dataset. The determination of cognitive status in PPMI involves evaluating different key domains: executive abilities, attention, memory, orientation, and language (Table 1). In the PPMI dataset, the “cogstate” variable categorizes the cognitive status of subjects into three categories: normal cognition, MCI, and dementia. Normal cognition is defined as individuals exhibiting intact cognitive functioning and typical cognitive abilities. Following the Level I MDS Task Force MCI definition, MCI is defined as having impairment in at least one cognitive domain, yet it does not have a noticeable impact on daily functioning. In contrast, dementia is diagnosed when there is impairment in functioning across multiple cognitive domains and a significant impact on daily life. More information on cognitive status evaluation in the PPMI dataset is available at https://www.ppmi-info.org. Over the course of the 5-year follow-up, we monitored changes in the “cogstate” variable for each patient. The CogState has demonstrated its sensitivity in recognizing cognitive impairment in numerous neurodegenerative disorders (Hammers et al., 2012; Lim et al., 2012). Using our search criteria, we identified 52 PD-UHC (PD patients who exhibited normal cognitive function at the baseline but progressed to MCI within 5 years) and 192 PD-SHC (PD patients who maintained healthy cognitive status within a 5-year time span from the baseline).

**TABLE 1:** The details of primary domains utilized for assessing cognitive status within the PPMI dataset (Wyman, 2018).

Neurological test	Cognitive function	Description
Letter Number Sequencing (LNS) (Tulsky and Ledbetter, 2000)	Attention and working memory	Evaluates the ability to sustain and direct attention, including instances of lapses.
Hopkins Verbal Learning Test Revised (HVLT-R) (Brandt, 1991)	Verbal learning and memory	Assesses registration, recall of recent events, new learning ability, and item retention.
Benton Judgment of Line Orientation (BJLO) (Benton and Varney, 1978)	Orientation and visuospatial judgment	Measures spatial orientation, estimating time, and forgetting appointments.
Symbol Digit Modalities Test (SDM) (Smith, 1973)	Executive abilities and processing speed	Examines reasoning ability, decision-making, instruction-following, and calculation difficulty.
Semantic Fluency (SF) (Kim et al., 2019)	Language and verbal ability	Identifies word-finding problems and issues with naming or comprehension.

In the PD-UHC group, the conversion from normal cognition to MCI occurred from 1 to 5 years after baseline with a mean of 3.23 (±1.32 years). PD patients showing any fluctuations in their cognitive status (i.e., reverters) were not included in the study. All PD Patients used in this study were diagnosed with idiopathic PD and did not have any other neurological disorders.

To balance the sample sizes between groups and to ensure that the developed classifier is only sensitive to the brain structural abnormality and unbiased to other baseline differences between PD-UHC and PD-SHC, we selected a subset of PD-SHC to match PD-UHC using propensity score matching. The matching process involved aligning the two groups based on criteria that included baseline items such as chronological age, education level, age of symptom onset, disease duration, UPDRS-III (total), UPDRS-III (total tremor), UPDRS-III (total rigidity), MoCA, depression, and anxiety. Propensity score matching was done using the *pymatch* package in Python.^1^ This package employs logistic regression models to generate propensity scores and facilitate the matching of the two groups. Following propensity score matching, 52 PD-SHC patients with similar clinical and demographic characteristics to 52 PD-UHC were selected for further analysis. This study was approved by the Health Research Ethics Board of the University of Manitoba.

### 2.2 MRI pre-processing and feature extraction

The MRI pre-processing was performed using the CAT12 Toolbox^2^ within the framework of Statistical Parametric Mapping Software Version 12.^3^ CAT12 is recognized as a prominent toolbox for voxel-based morphometry (VBM) analysis (Farokhian et al., 2017). Furthermore, it allows for the performance of regional analyses via region-based morphometry (RBM). In this scenario, CAT12 utilizes spatial registration parameters from voxel-based processing to align volumetric atlases onto individual brains. This feature facilitates the determination of volumetric measures, including regional gray matter volume, for each region of interest (ROI) within its native space. Further details regarding the CAT12 pipeline can be found in Gaser et al. (2022). The MRI pre-processing was performed using the default settings in CAT12. By utilizing the “Estimate mean values inside ROI” function in CAT12, we extracted 170 volumetric data for gray matter (GM) based on the Automated Anatomical Labeling Atlas 3 (AAL3) (Rolls et al., 2020). These data were used as brain features in our prediction model (number of features = 170). To control for the effect of brain size, the ROI volumes of each subject from the AAL3 atlas were divided by the respective total intracranial volume (TIV). TIV volumes were also calculated using the CAT12 toolbox.

### 2.3 Classification and validation

Our prediction model was developed using SVM and implemented in Python programming language (version 3.9.12) with the Scikit-Learn package (version 1.1.1). Of 104 samples (52 PD-UHC and 52 PD-SHC), we randomly selected 75% of the data as a training set (39 PD-UHC and 39 PD-SHC) to create a model and the remaining 25% of the data as an independent test set (13 PD-UHC and 13 PD-SHC). The prediction accuracy and adjusting the hyperparameters of SVM within the training set were computed using a 10-fold cross-validation strategy. The parameter grid was defined, consisting of the kernel type (linear or radial basis function), regularization parameter (C), and the kernel coefficient (gamma). The range of the C and gamma was set to 2 to the power of −10 up to 10 with 0.5 intervals. The GridSearchCV function with 10-fold cross-validation was used to conduct grid search to determine the best-performing model, which was reported by the highest mean accuracy from the cross-validation sets. The entire training set (*N* = 78) along with the optimal kernel and hyperparameters was used to build the final prediction model, which was then applied to independent test set (*N* = 26).

To ascertain which brain regions are most influential in the classification tasks, we employed the SHAP analysis technique. The Shapley value is a key element of cooperative game theory and is widely employed in predictive modeling. Shapley values represent the individual contribution of a specific variable to a model’s prediction, and they show how important each variable is relative to the overall prediction (Merrick and Taly, 2020). The Shapley values were extracted using the SHAP package in Python.^4^ The predictor object, comprising the finalized model and the test dataset, was used to calculate the Shapley values of each sample with 10,000 Monte Carlo simulations. The absolute Shapley values were then averaged across all the samples, giving an overall assessment of the global Shapley contribution of each variable, as described in Harvey et al. (2022).

### 2.4 Statistical analysis

All statistical analyses were performed using Python. Baseline demographic, clinical variables, and brain GM volumes between two groups were examined using two-sample *t*-tests. Categorical variables underwent analysis through Chi-square tests. The accuracy (ACC), sensitivity (SEN), specificity (SPE), and area under the curve (AUC) metrics were used to report the classification performance. The *P*-values were adjusted using the false discovery rate (FDR) strategy. A significance level of *P* < 0.05 was used to determine the statistical significance of all tests.

## 3 Results

### 3.1 Clinical demographics

As intended by propensity score matching procedure, the demographic characteristics and clinical scores were not significantly different between PD-UHC and PD-SHC patients (Table 2).

**TABLE 2:** Clinical and demographic features of PD patients included in this study, categorized by cognitive status.

Characteristics	PD-SNC	PD-UNC	PD-SNC	PD-UNC	PD-SNC	PD-UNC
	**Baseline**		**At time of conversion**		**Last record**	
*N* (male %)	52 (71.15%)	52 (71.15%)	52 (71.15%)	52 (71.15%)	52 (71.15%)	52 (71.15%)
Demographics	Mean (SD)	Mean (SD)	Mean (SD)	Mean (SD)	Mean (SD)	Mean (SD)
Age, years	61.98 (8.27)	64.33 (7.81)	n.a.	67.56 (7.93)	66.98 (8.27)	69.33 (7.80)
Onset age, years	60.16 (8.24)	62.4 (7.91)	n.a.	n.a.	n.a.	n.a.
Education, years	15.58 (2.83)	15.06 (2.52)	n.a.	n.a.	n.a.	n.a.
Disease duration, months	5.88 (6.49)	6.18 (6.72)	n.a.	44.94 (16.12)	65.88 (6.49)	66.17 (6.72)
**Motor symptoms**						
UPDRS-III (total)	31.67 (11.59)	33.18 (13.31)	n.a.	61.77 (18.62)	46.44 (12.03)	63.30 (21.95)***
UPDRS-III (total rigidity)	3.60 (2.46)	3.46 (2.60)	n.a.	5.66 (3.12)	6.38 (3.30)	6.76 (3.72)
UPDRS-III (total tremor)	4.52 (2.84)	5.10 (3.84)	n.a.	6.7 (5.3)	5.96 (4.42)	6.65 (5.17)
**Cognitive symptoms**						
MoCA	26.94 (2.06)	26.56 (2.93)	n.a.	24.51 (3.95)	27.30 (2.38)	24.15 (4.45)***
LNS	10.69 (2.64)	9.63 (2.72)	n.a.	8.98 (3.17)	10.38 (2.59)	8.67 (2.99)**
BJLOT	12.82 (2.20)	12.30 (2.47)	n.a.	11.32 (3.05)	12.53 (2.64)	11.09 (2.94)*
SDM	41.47 (9.13)	38.78 (8.86)	n.a.	34.19 (11.22)	44.13 (11.64)	32.84 (12.54)***
SFT	48.25 (10.66)	45.88 (9.69)	n.a.	44.38 (10.80)	50.00 (12.44)	43.26 (11.82)*
Epworth sleepiness scale	5.31 (3.03)	5.17 (3.52)	n.a.	6.87 (4.22)	6.78 (4.14)	8.82 (5.38)*
**Mood**						
Anxiety	63.54 (17.13)	68.25 (17.62)	n.a.	70.0 (19.30)	59.94 (17.60)	71.88 (19.25)**
GDS	1.88 (1.85)	2.52 (2.40)	n.a.	3.36 (2.72)	1.98 (2.40)	3.75 (2.74)**

BJLOT, Benton Judgment of Line Orientation Score; GDS, Geriatric Depression Scale; HC, healthy control; LNS, Letter Number Sequencing; MoCA, Montreal Cognitive Assessment; PD, Parkinson’s disease; SDM, Symbol Digit Modalities Test; SFT, Semantic Fluency Total Score; UPDRS, Unified Parkinson Disease Rating Scale; N, number of subjects; n.a., not available. The significance levels were presented as outcomes of a Chi-square test for categorical variables and a *t*-test for continuous variables comparing the PD-UNC and PD-SNC groups, following FDR correction for multiple comparisons (*P < 0.05, **P < 0.001, ***P < 0.0001).

### 3.2 Classification performance within the training cohort

**FIGURE 1:**
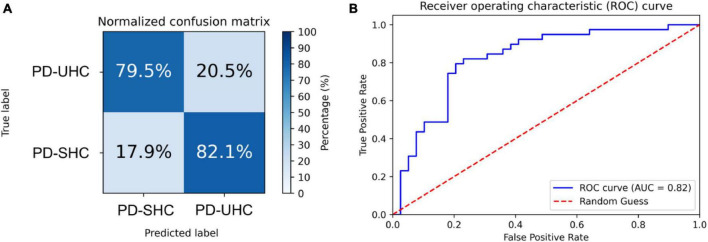
**(A)** Normalized confusion matrix and **(B)** receiver operating characteristic plot for predicting cognitive impairment within the training set using 10-fold cross-validation.

The hyperparameters of SVM were determined through grid search within the training set, with the kernel being a radial basis function (RBF), the regularization parameter (C) being 32, and the parameter for the RBF kernel in SVM (gamma) being 0.0039. An accuracy of 80.76% was achieved in differentiating between PD-UHC and PD-SHC, with a sensitivity of 82.05%, a specificity of 79.48% and AUC of 0.82. Figure 1 displays the confusion matrix and ROC for the training set, which was obtained by implementing a 10-fold cross validation technique.

### 3.3 Classification performance on the hold-out set

**FIGURE 2:**
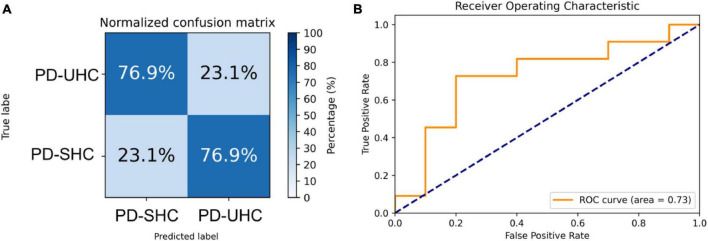
Representation of the **(A)** normalized confusion matrix and **(B)** the receiver operating characteristic plot for the prediction of cognitive impairment in the hold-out set.

When tested on the hold-out set of 13 PD-UHC and 13 PD-SHC, the proposed model achieved an accuracy of 76.92%. The sensitivity, specificity and AUC of the results were 76.92%, 76.92%, and 0.73%, respectively. The confusion matrix and ROC for the hold-out set are presented in Figure 2.

### 3.4 Predictive variables for cognitive impairment outcome

**FIGURE 3:**
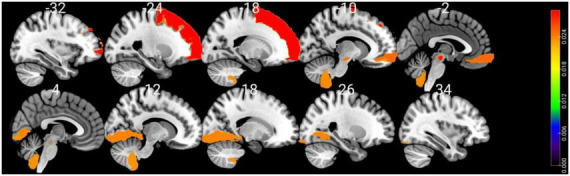
Visualizing the top 10 brain regions involving in SVM machine learning prediction of cognitive impairment using structural MRI data. The color bar stands for Shapley values.

Shapley values of the top 10 regions are summarized in Table 3 and visualized on the AAL3 template (Figure 3). The complete table is provided in the Supplementary material.

**TABLE 3:** The list of the top 10 brain regions determined by Shapley for MCI prediction in PD.

Abbreviation in AAL3 atlas	Brain region	Shapley value
lSFG	Left superior frontal gyrus-dorsolateral	0.0289
lRedN	Left red nucleus	0.0281
lSNpr	Left substantia nigra-pars reticulata	0.0251
lPFCventmed	Left superior frontal gyrus-medial orbital	0.0250
rVTA	Right ventral tegmental area	0.0249
RapheD	Raphe nucleus-dorsal	0.0241
rLING	Right lingual gyrus	0.0240
RapheM	Raphe nucleus-median	0.0239
lCER9	Left lobule IX of cerebellar hemisphere	0.0237
rCER9	Right lobule IX of cerebellar hemisphere	0.0235

## 4 Discussion

In this study, we developed a predictive model using structural T1w MRI and SVM to classify PD-UHC vs. PD-SHC with high accuracy (AUC = 0.73), sensitivity (76.92%), and specificity (76.92%) on the hold-out set. The identification of MCI status in PD has become a necessary area of research, as it can give insight into the mechanisms of cognitive decline in PD (Sun et al., 2022). In previous studies, addressing cognitive decline in PD, supervised machine-learning approaches coupled with neuroimaging data have been used to discriminate between PD-MCI and PD-HC (Cho, 2019; Zhang et al., 2020, 2021; Shin et al., 2021). A notable difference in these investigations from the present study is that they were carried out on PD patients exhibiting stable cognitive functioning (PD-SHC) and those who had been already identified with MCI at the baseline (PD-MCI). In contrast, we used a supervised machine learning approach to distinguish PD-HC patients from matched PD patients who progressed to MCI years after the initial assessment, and to identify the structural brain differences between the two groups.

To date, only a few studies have explored the potential of machine learning techniques to predict cognitive outcomes in PD before clinical symptoms arise, and these studies primarily utilize clinical variables for this purpose (Smith et al., 2021; Harvey et al., 2022). For instance, a predictive model trained on clinical and biological parameters exhibited robust accuracy in predicting cognitive impairment and maintaining normal cognition over an 8-year follow-up period, with an AUC of 0.86 (Harvey et al., 2022). The relevance of clinical metrics, such as anxiety and olfactory impairment, as well as biological markers like DNA methylation, is also highlighted in this study, indicating their possibility of being used as indicators for cognitive outcomes in PD (Harvey et al., 2022). The efficacy of using cortical structure was also assessed in predicting cognitive performance in PD patients, at least 3 years before the onset of MCI symptoms, yielding an AUC of 0.72 (Smith et al., 2021). In this pre-print (not peer-reviewed) study, the predictive model was further enhanced by incorporating clinical variables and structural imaging data, leading to an improved AUC of 0.85 (Smith et al., 2021). Interestingly, they could achieve AUC of 0.81 only with clinical variables without neuroimaging data suggesting their smaller contribution to the decision-making process of the prediction model (Smith et al., 2021). To prevent that our model is dictated by baseline clinical characteristics (e.g., lower MoCA scores), we selected a subset of PD-SHC patients that were matched with PD-UHC patients in terms of age, gender, and clinical characteristics (Table 2).

Using SHAP analysis, we ranked the contribution of brain regions (in terms of GM volume) on our prediction model. Top-contributing brain regions located in the frontal, occipital, and cerebellar regions as well as the midbrain (Table 3). Particularly, the left superior frontal gyrus-dorsolateral was shown as the top brain region, which is directly associated with cognitive executive functions such as working memory and decision-making (Li et al., 2013). The abnormality of the dorsolateral prefrontal cortex has well been documented in PD-MCI (Nagano-Saito et al., 2014; Mihaescu et al., 2019), and the electrical stimulation therapies on this region resulted in significant improvement in PD cognition (Randver, 2018; Beheshti and Ko, 2021).

Interestingly, several regions in the midbrain area (e.g., the right ventral tegmental area and the raphe nucleus-dorsal) were detected as highly important regions in our prediction model. The primary pathological feature of PD is the deterioration of neurons in the substantia nigra, leading to the gradual death of these cells, with up to 70% loss over time (Caminero and Cascella, 2019). While the midbrain region is commonly known with movement and coordination, it plays a pivotal role in transmitting essential information for vision and hearing processes (Caminero and Cascella, 2019). It also serves as a key area for functions related to reward cognition (e.g., motivational salience and associative learning), consciousness, and sleep (Caminero and Cascella, 2019). Additionally, there may be a connection between hyperechogenicity of the substantia nigra and a slight decrease in performance on the word list delayed recall test (Yilmaz et al., 2016), which aligns with previous findings of memory issues in early PD (Aarsland et al., 2009; Broeders et al., 2013).

Our machine-learning analysis also suggests that the cerebellum is one of the key brain regions associated with early cognitive deterioration in PD (Table 3). Historically, the cerebellum has been viewed as playing a role in the management of voluntary movement, motor learning, and balance (Wu and Hallett, 2013). In the context of PD, even though cerebellar abnormalities have conventionally been associated with tremors (Zhong et al., 2022) and gait disturbances (Wu and Hallett, 2013), recent studies have found connections between the cerebellum and cognitive decline (Wu and Hallett, 2013). Identifying Lobule IX of the cerebellum as a top brain region associated with cognitive decline is also consistent with other studies that have documented associations between Lobule IX of the cerebellum and the behavioral components of cognition and emotions in PD (Azizi, 2021). We also performed an independent *t*-test comparing GM volumetric features between two groups. This analysis indicated a significant group difference in only one region: the Right Lobule III of the cerebellar hemisphere [*t*(102) = 3.38, *P* = 0.041, FDR corrected]. This finding suggests that the classifier we developed is not solely influenced by a single regional variation. Instead, it underscores the importance of the overall pattern of GM atrophy across a wide network of brain regions in predicting MCI in PD.

Cross-sectional studies have reported a prevalence of around 25.8% of MCI at the time of PD diagnosis (Aarsland et al., 2010). Longitudinal studies have previously shown 20%–25% prevalence of MCI when first diagnosed with PD which increased to 40%–50% after 5 years of monitoring (Domellöf et al., 2015; Lawson et al., 2017; Pedersen et al., 2017). The dementia prevalence also increases as disease duration increases: 17% after 5 years of diagnosis (Williams-Gray et al., 2009), 46% after 10 years (Williams-Gray et al., 2013), and 83% after 20 years (Hely et al., 2008). On the contrary, the PPMI cohort that we have downloaded (who met our inclusion criteria) shows much less incidence of MCI conversion over 5 years (21.3%). This discrepancy may stem from the use of different diagnostic criteria, positive shift toward PD awareness (earlier identification), and/or differences in study volunteer recruitment strategies. For example, the PPMI patients that we have included were much younger (63 ± 8 years old) than previous studies (71 ± 7 years old), and older age at diagnosis is a known risk factor for cognitive decline in PD (Domellöf et al., 2015; Anang et al., 2017; Pedersen et al., 2017).

The biggest limitation of the current study is the small sample size, which was constrained by the number of PD-UHC. Our findings need to be validated by additional studies with larger sample sizes, particularly in relation to the most important brain regions linked to the early stages of cognitive decline in PD. Furthermore, the tracking interval for our samples was limited to 5 years, preventing us from following the cognitive status of our PD-SHC patients over a longer period, such as 8 years (Aarsland et al., 2003).

Another significant constraint is the absence of longitudinally acquired MRI scans. Anticipated completion of data collection in the near future for the PPMI is expected to address this limitation by providing a larger and longitudinal dataset. This expanded dataset may encompass a substantial number of patients transitioning from cognitively healthy states to MCI or dementia, using diverse brain imaging modalities such as resting state functional MRI and Diffusion tensor imaging. The prospective nature of this data will enable us to develop a more comprehensive model for predicting cognitive decline in PD.

## 5 Conclusion

In this study, we used a SVM along with baseline structural MRI data to construct a model that accurately predicted cognitive impairment and preserved normal cognition in diagnosed PD cases from the PPMI over a 5-year follow-up period. This prediction was driven by baseline MRI features from two PD groups (e.g., PD-SHC and PD-UHC) that were similar in terms of their baseline clinical and demographic characteristics. Our analysis highlighted a discernible pattern of GM alterations between these two groups, predominantly localized in the frontal, midbrain, occipital, and cerebellum regions.

## Data availability statement

The original contributions presented in this study are included in the article/Supplementary material, further inquiries can be directed to the corresponding author.

## Ethics statement

The study received approval from the Health Research Ethics Board of the University of Manitoba. The data employed in this study was sourced from the Parkinson Progression Markers Initiative (PPMI) dataset. Prior to the initiation of the study, all PPMI sites underwent scrutiny and were approved by an ethical standards committee. All participants provided written consent before their involvement. For more information on PPMI protocol approvals, patient consents, and registrations, please refer to: https://www.ppmi-info.org/.

## Author contributions

IB: Conceptualization, Data curation, Formal analysis, Investigation, Methodology, Validation, Visualization, Writing – original draft, Writing – review & editing. JK: Conceptualization, Funding acquisition, Investigation, Project administration, Resources, Supervision, Writing – review & editing.

## Footnotes

1
https://github.com/benmiroglio/pymatch

2
http://www.neuro.uni-jena.de/cat/

3
https://www.fil.ion.ucl.ac.uk/spm/software/spm12/

4
https://shap.readthedocs.io/en/latest/
