# Supplementary material for: Predicting the occurrence of mild cognitive impairment in Parkinson’s disease using structural MRI data
Source: Front Neurosci. 2024 Apr 18;18:1375395. doi: 10.3389/fnins.2024.1375395 (PMC11063344; doi:10.3389/fnins.2024.1375395)
Supplement: Supplementary file 1 [file Data_Sheet_1.docx]

Supplementary Information


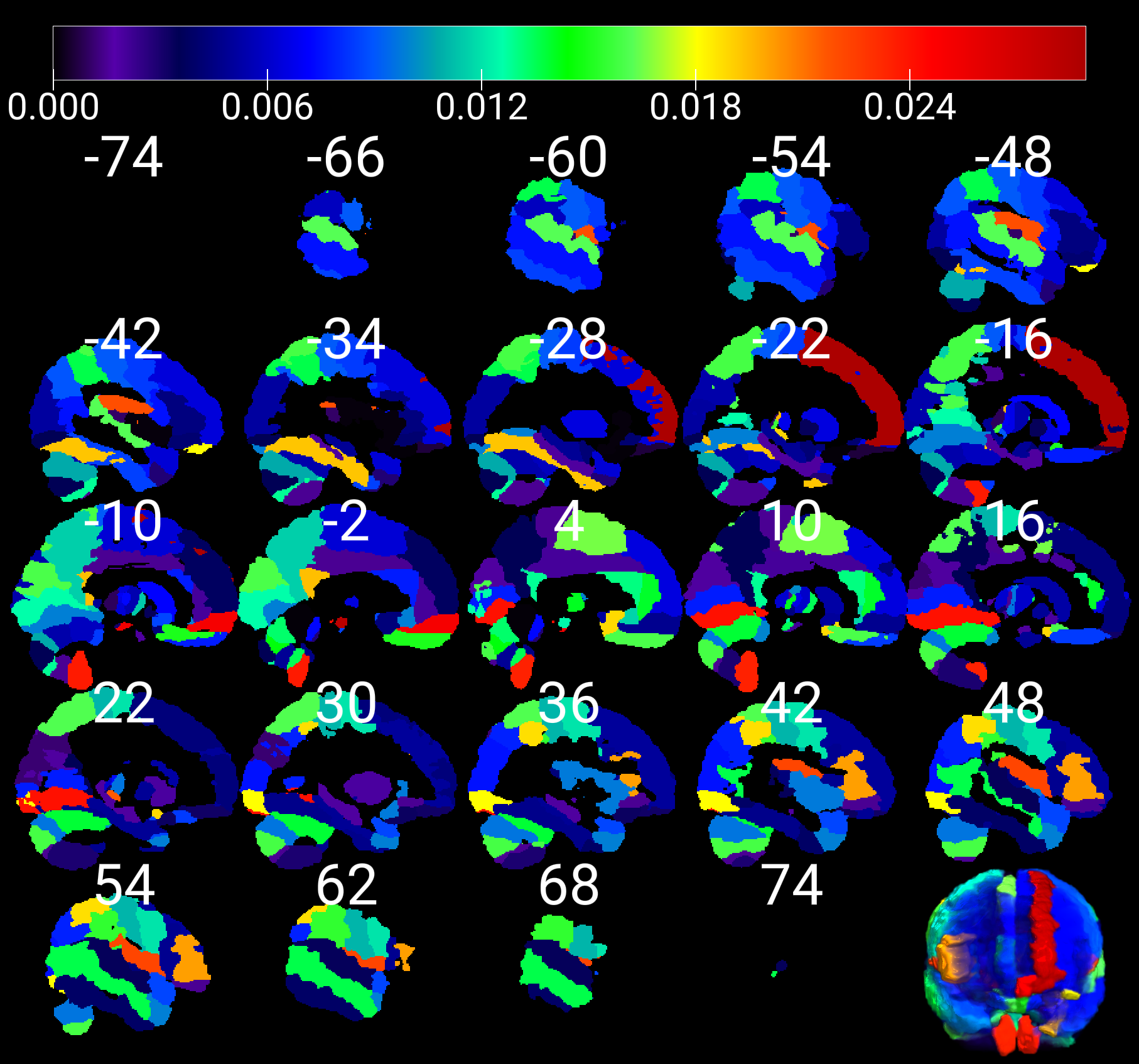


**Supplementary Figure 1:** Visualizing all brain regions involving in SVM machine learning prediction of cognitive impairment using structural MRI data. The color bar stands for Shapley values.

**Supplementary Table 1:** Summary of Shapley values for all brain regions in predicting MCI in PD.

| Region number | Abbreviation in AAL3 atlas | Brain region | Shapley value |
| --- | --- | --- | --- |
| 3 | lSFG | Left Superior frontal gyrus-dorsolateral | 0.028925305 |
| 165 | lRedN | Left Red nucleus | 0.0281241 |
| 163 | lSNpr | Left Substantia nigra-pars reticulata | 0.025134617 |
| 21 | lPFCventmed | Left Superior frontal gyrus-medial orbital | 0.025022005 |
| 160 | rVTA | Right Ventral tegmental area | 0.024940472 |
| 169 | RapheD | Raphe nucleus-dorsal | 0.024127575 |
| 52 | rLING | Right Lingual gyrus | 0.024024869 |
| 170 | RapheM | Raphe nucleus-median | 0.023913023 |
| 109 | lCER9 | Left Lobule IX of cerebellar hemisphere | 0.023666987 |
| 110 | rCER9 | Right Lobule IX of cerebellar hemisphere | 0.023521308 |
| 14 | rROL | Right Rolandic operculum | 0.022231888 |
| 13 | lROL | Left Rolandic operculum | 0.021904517 |
| 140 | rtLGN | Right Lateral geniculate | 0.021527095 |
| 10 | rIFGtriang | Right Inferior frontal gyrus-triangular part | 0.020057348 |
| 149 | ltPuI | Left Pulvinar inferior | 0.019463868 |
| 39 | lPCC | Left Posterior cingulate gyrus | 0.01935358 |
| 59 | lFFG | Left Fusiform gyrus | 0.019319333 |
| 139 | ltLGN | Left Lateral geniculate | 0.018920501 |
| 18 | rOLF | Right Olfactory cortex | 0.018846306 |
| 66 | rIPG | Right Inferior parietal gyrus-excluding supramarginal and angular gyri | 0.018747309 |
| 31 | lOFClat | Left Lateral orbital gyrus | 0.018041656 |
| 58 | rIOG | Right Inferior occipital gyrus | 0.017997333 |
| 16 | rSMA | Right Supplementary motor area | 0.016591853 |
| 85 | lSTG | Left Superior temporal gyrus | 0.016271472 |
| 64 | rSPG | Right Superior parietal gyrus | 0.016104139 |
| 49 | lCUN | Left Cuneus | 0.015987324 |
| 24 | rREC | Right Gyrus rectus | 0.015971401 |
| 98 | rCERCRU2 | Right Crus II of cerebellar hemisphere | 0.015872222 |
| 63 | lSPG | Left Superior parietal gyrus | 0.015863719 |
| 143 | ltPuA | Left Pulvinar anterior | 0.015692591 |
| 68 | rSMG | Right SupraMarginal gyrus | 0.015442384 |
| 23 | lREC | Left Gyrus rectus | 0.015018018 |
| 117 | VER7 | Lobule VII of vermis | 0.014306927 |
| 136 | rtMDm | Right Mediodorsal medial magnocellular | 0.014075473 |
| 154 | rACCpre | Right Anterior cingulate cortex-pregenual | 0.013901815 |
| 104 | rCER6 | Right Lobule VI of cerebellar hemisphere | 0.013874944 |
| 102 | rCER4_5 | Right Lobule IV-V of cerebellar hemisphere | 0.013868545 |
| 90 | rMTG | Right Middle temporal gyrus | 0.013609828 |
| 65 | lIPG | Left Inferior parietal gyrus-excluding supramarginal and angular gyri | 0.013587778 |
| 162 | rSNpc | Right Substantia nigra-pars compacta | 0.01353399 |
| 119 | VER9 | Lobule IX of vermis | 0.013520468 |
| 126 | rtVA | Right Ventral anterior | 0.013474299 |
| 116 | VER6 | Lobule VI of vermis | 0.013234515 |
| 156 | rACCsup | Right Anterior cingulate cortex-supracallosal | 0.012999173 |
| 40 | rPCC | Right Posterior cingulate gyrus | 0.012798823 |
| 105 | lCER7b | Left Lobule VIIB of cerebellar hemisphere | 0.01277849 |
| 47 | lCAL | Left Calcarine fissure and surrounding cortex | 0.012577464 |
| 166 | rRedN | Right Red nucleus | 0.012516639 |
| 2 | rPreCG | Right Precentral gyrus | 0.012068877 |
| 152 | rACCsub | Right Anterior cingulate cortex-subgenual | 0.011650114 |
| 71 | lPCUN | Left Precuneus | 0.011524176 |
| 125 | ltVA | Left Ventral anterior | 0.011437525 |
| 62 | rPoCG | Right Postcentral gyrus | 0.01094888 |
| 95 | lCERCRU1 | Left Crus I of cerebellar hemisphere | 0.010812475 |
| 124 | rtLP | Right Lateral posterior | 0.010070133 |
| 100 | rCER3 | Right Lobule III of cerebellar hemisphere | 0.010033541 |
| 122 | rtAV | Right Thalamus-Anteroventral Nucleus | 0.009996065 |
| 51 | lLING | Left Lingual gyrus | 0.009898627 |
| 151 | lACCsub | Left Anterior cingulate cortex-subgenual | 0.009841338 |
| 92 | rTPOmid | Right Temporal pole: middle temporal gyrus | 0.009837754 |
| 34 | rINS | Right Insula | 0.009795969 |
| 96 | rCERCRU1 | Right Crus I of cerebellar hemisphere | 0.009633369 |
| 130 | rtVPL | Right Ventral posterolateral | 0.009596177 |
| 61 | lPoCG | Left Postcentral gyrus | 0.009045128 |
| 141 | ltMGN | Left Medial Geniculate | 0.009036005 |
| 69 | lANG | Left Angular gyrus | 0.008925534 |
| 157 | lNacc | Left Nucleus accumbens | 0.008638295 |
| 88 | rTPOsup | Right Temporal pole: superior temporal gyrus | 0.00862883 |
| 159 | lVTA | Left Ventral tegmental area | 0.008579082 |
| 93 | lITG | Left Inferior temporal gyrus | 0.008523602 |
| 99 | lCER3 | Left Lobule III of cerebellar hemisphere | 0.008445516 |
| 26 | rOFCmed | Right Medial orbital gyrus | 0.008440642 |
| 1 | lPreCG | Left Precentral gyrus | 0.008346962 |
| 111 | lCER10 | Left Lobule X of cerebellar hemisphere | 0.008326109 |
| 70 | rANG | Right Angular gyrus | 0.007827972 |
| 25 | lOFCmed | Left Medial orbital gyrus | 0.00770236 |
| 48 | rCAL | Right Calcarine fissure and surrounding cortex | 0.007679926 |
| 155 | lACCsup | Left Anterior cingulate cortex-supracallosal | 0.007662824 |
| 7 | lIFGoperc | Left Inferior frontal gyrus-opercular part | 0.007531681 |
| 56 | rMOG | Right Middle occipital gyrus | 0.007467663 |
| 89 | lMTG | Left Middle temporal gyrus | 0.007441369 |
| 150 | rtPuI | Right Pulvinar inferior | 0.007343577 |
| 137 | ltMDl | Left Mediodorsal lateral parvocellular | 0.007245659 |
| 114 | VER3 | Lobule III of vermis | 0.007231155 |
| 75 | lCAU | Left Caudate nucleus | 0.007137254 |
| 147 | ltPuL | Left Pulvinar lateral | 0.006922888 |
| 133 | ltRe | Left Reuniens | 0.006782353 |
| 138 | rtMDl | Right Mediodorsal lateral parvocellular | 0.006743853 |
| 129 | ltVPL | Left Ventral posterolateral | 0.006654208 |
| 20 | rSFGmedial | Right Superior frontal gyrus-medial | 0.006507175 |
| 77 | lPUT | Left Lenticular nucleus-Putamen | 0.006484669 |
| 135 | ltMDm | Left Mediodorsal medial magnocellular | 0.006413308 |
| 5 | lMFG | Left Middle frontal gyrus | 0.006394839 |
| 123 | ltLP | Left Lateral posterior | 0.006324468 |
| 131 | ltIL | Left Intralaminar | 0.006282123 |
| 15 | lSMA | Left Supplementary motor area | 0.006250767 |
| 87 | lTPOsup | Left Temporal pole: superior temporal gyrus | 0.006231995 |
| 73 | lPCL | Left Paracentral lobule | 0.006200508 |
| 146 | rtPuM | Right Pulvinar medial | 0.006072921 |
| 128 | rtVL | Right Ventral lateral | 0.006051128 |
| 101 | lCER4_5 | Left Lobule IV-V of cerebellar hemisphere | 0.005813811 |
| 67 | lSMG | Left SupraMarginal gyrus | 0.005787962 |
| 127 | ltVL | Left Ventral lateral | 0.005494959 |
| 103 | lCER6 | Left Lobule VI of cerebellar hemisphere | 0.005376457 |
| 53 | lSOG | Left Superior occipital gyrus | 0.005375732 |
| 76 | rCAU | Right Caudate nucleus | 0.005236791 |
| 55 | lMOG | Left Middle occipital gyrus | 0.005086933 |
| 158 | rNacc | Right Nucleus accumbens | 0.005027406 |
| 6 | rMFG | Right Middle frontal gyrus | 0.005017026 |
| 44 | rPHG | Right Parahippocampal gyrus | 0.004904699 |
| 121 | ltAV | Left Thalamus-Anteroventral Nucleus | 0.004894863 |
| 118 | VER8 | Lobule VIII of vermis | 0.004894329 |
| 32 | rOFClat | Right Lateral orbital gyrus | 0.004889274 |
| 80 | rPAL | Right Lenticular nucleus-Pallidum | 0.00484583 |
| 41 | lHIP | Left Hippocampus | 0.004829331 |
| 22 | rPFCventmed | Right Superior frontal gyrus-medial orbital | 0.004783753 |
| 112 | rCER10 | Right Lobule X of cerebellar hemisphere | 0.004783161 |
| 8 | rIFGoperc | Right Inferior frontal gyrus-opercular part | 0.004753191 |
| 60 | rFFG | Right Fusiform gyrus | 0.00468542 |
| 120 | VER10 | Lobule X of vermis | 0.004488158 |
| 9 | lIFGtriang | Left Inferior frontal gyrus-triangular part | 0.00442916 |
| 132 | rtIL | Right Intralaminar | 0.004370314 |
| 84 | rHES | Right Heschls gyrus | 0.00434019 |
| 4 | rSFG | Right Superior frontal gyrus-dorsolateral | 0.004334799 |
| 17 | lOLF | Left Olfactory cortex | 0.004293757 |
| 45 | lAMYG | Left Amygdala | 0.004225292 |
| 11 | lIFGorb | Left IFG pars orbitalis | 0.004040227 |
| 94 | rITG | Right Inferior temporal gyrus | 0.003807074 |
| 19 | lSFGmedial | Left Superior frontal gyrus-medial | 0.003772375 |
| 57 | lIOG | Left Inferior occipital gyrus | 0.003548053 |
| 72 | rPCUN | Right Precuneus | 0.003532793 |
| 97 | lCERCRU2 | Left Crus II of cerebellar hemisphere | 0.00350439 |
| 86 | rSTG | Right Superior temporal gyrus | 0.003386842 |
| 79 | lPAL | Left Lenticular nucleus-Pallidum | 0.003355652 |
| 30 | rOFCpost | Right Posterior orbital gyrus | 0.003308637 |
| 144 | rtPuA | Right Pulvinar anterior | 0.003227694 |
| 28 | rOFCant | Right Anterior orbital gyrus | 0.003225641 |
| 161 | lSNpc | Left Substantia nigra-pars compacta | 0.003012762 |
| 108 | rCER8 | Right Lobule VIII of cerebellar hemisphere | 0.002980791 |
| 91 | lTPOmid | Left Temporal pole: middle temporal gyrus | 0.00283045 |
| 113 | VER1_2 | Lobule I-II of vermis | 0.002804022 |
| 142 | rtMGN | Right Medial Geniculate | 0.002248511 |
| 164 | rSNpr | Right Substantia nigra-pars reticulata | 0.002185675 |
| 153 | lACCpre | Left Anterior cingulate cortex-pregenual | 0.002078161 |
| 38 | rMCC | Right Middle cingulate &amp; paracingulate gyri | 0.002017614 |
| 78 | rPUT | Right Lenticular nucleus-Putamen | 0.001930512 |
| 42 | rHIP | Right Hippocampus | 0.001631168 |
| 50 | rCUN | Right Cuneus | 0.001606006 |
| 43 | lPHG | Left Parahippocampal gyrus | 0.001556338 |
| 12 | rIFGorb | Right IFG pars orbitalis | 0.001495246 |
| 107 | lCER8 | Left Lobule VIII of cerebellar hemisphere | 0.001492444 |
| 37 | lMCC | Left Middle cingulate &amp; paracingulate gyri | 0.001491599 |
| 106 | rCER7b | Right Lobule VIIB of cerebellar hemisphere | 0.001487608 |
| 148 | rtPuL | Right Pulvinar lateral | 0.001426816 |
| 74 | rPCL | Right Paracentral lobule | 0.001423143 |
| 54 | rSOG | Right Superior occipital gyrus | 0.001145513 |
| 83 | lHES | Left Heschls gyrus | 0.001015716 |
| 145 | ltPuM | Left Pulvinar medial | 0.000948037 |
| 27 | lOFCant | Left Anterior orbital gyrus | 0.000640873 |
| 33 | lINS | Left Insula | 0.000326587 |
| 29 | lOFCpost | Left Posterior orbital gyrus | 0.000213991 |
| 115 | VER4_5 | Lobule IV-V of vermis | 0.000173987 |
| 35 | lACC | Left Anterior cingulate &amp; paracingulate gyri | 0.000139593 |
| 81 | lTHA | Left Thalamus | 0.000137489 |
| 168 | rLC | Right Locus coeruleus | 0.000135842 |
| 167 | lLC | Left Locus coeruleus | 0.000135749 |
| 134 | rtRe | Right Reuniens | 0.000135556 |
| 36 | rACC | Right Anterior cingulate &amp; paracingulate gyri | 0.000135168 |
| 82 | rTHA | Right Thalamus | 0.000134092 |
| 46 | rAMYG | Right Amygdala | 7.32947E-05 |
